# Identification of a methylomics-associated nomogram for predicting overall survival of stage I–II lung adenocarcinoma

**DOI:** 10.1038/s41598-021-89429-4

**Published:** 2021-05-11

**Authors:** Heng Wang, Chuangye Wei, Peng Pan, Fengfeng Yuan, Jiancheng Cheng

**Affiliations:** 1 Department of Cardiothoracic Surgery, Zhengzhou Central Hospital Affiliated To Zhengzhou University, Zhengzhou, 450000 China; 2 Department of Thoracic Surgery, Zhengzhou Central Hospital Affiliated To Zhengzhou University, Zhengzhou, 450000 China; 3 Department of Mood Disorders, Nankai University Affiliated Anding Hospital, Tianjin Mental Health Center, Mental Health Teaching Hospital, Tianjin Medical University, Tianjin, 300222 China

**Keywords:** Cancer, Computational biology and bioinformatics, Genetics, Oncology

## Abstract

The aim of this paper was to identify DNA methylation based biomarkers for predicting overall survival (OS) of stage I–II lung adenocarcinoma (LUAD) patients. Methylation profile data of patients with stage I–II LUAD from The Cancer Genome Atlas (TCGA) database was used to determine methylation sites-based hallmark for stage I–II LUAD patients’ OS. The patients were separated into training and validation datasets by using median risk score as cutoff. Univariate Cox, least absolute shrinkage and selection operator (LASSO) and multivariate Cox analyses were employed to develop a DNA methylation signature for OS of patients with stage I–II LUAD. As a result, an 11-DNA methylation signature was determined to be critically associated with the OS of patients with stage I–II LUAD. Analysis of receiver operating characteristics (ROC) suggested a high prognostic effectiveness of the 11-DNA methylation signature in patients with stage I–II LUAD (AUC at 1, 3, 5 years in training set were (0.849, 0.879, 0.831, respectively), validation set (0.742, 0.807, 0.904, respectively), entire TCGA dataset (0.747, 0.818, 0.870, respectively). Kaplan–Meier survival analyses exhibited that survival was significantly longer in the low-risk cohort compared to the high-risk cohort in the training dataset (P = 7e − 07), in the validation dataset (P = 1e − 08), and in the all-cohort dataset (P = 6e − 14). In addition, a nomogram was developed based on molecular factor (methylation risk score) as well as clinical factors (age and cancer status) (AUC at 1, 3, 5 years entire TCGA dataset were 0.770, 0.849, 0.979, respectively). The result verified that our methylomics-associated nomogram had a strong robustness for predicting stage I–II LUAD patients’ OS. Furthermore, the nomogram combined clinical and molecular factors to determine an individualized probability of recurrence for patients with stage I–II LUAD, which stood for a major advance in the field of personalized medicine for pulmonary oncology. Collectively, we successfully identified a DNA methylation biomarker and a DNA methylation-based nomogram to predict the OS of patients with stage I–II LUAD.

## Introduction

Lung cancer is the most common type of cancer in terms of cancer-related death worldwide^1^. Non-small-cell lung cancers (NSCLCs) accounts for roughly 85% of all lung cancers based on pathomorphology^2^. Importantly, 35% of the NSCLCs are diagnosed as LUAD. In spite of the improvement of treatment, 5-year survival rate of NSCLC is poor^3^. Nearly 30% of stage I NSCLC patients would undergo recurrent disease, and many of them would die due to cancer recurrence after surgical therapy^3,4^. Therefore, exploring novel prognostic hallmarks could assist clinicians in prognostic evaluations and therapeutic selection for early stage LUAD patients.

Growing researches showed that specific molecules could function as prognostic markers for lung cancer. For example, Wang et al. found potential diagnostic and prognostic biomarkers of circular RNAs for lung cancer in China^5^. Ning et al. suggested that CPSF3 was a promising prognostic biomarker and predicted recurrence of NSCLC^6^. Liu et al. revealed lncRNA SLC16A1-AS1 as a novel prognostic hallmark in NSCLC^7^. Zhang et al. identified six metabolic genes as potential biomarkers for LUAD^8^. Meanwhile, emerging studies have demonstrated that epigenetics plays a critical role in the initiation, progression, therapeutic response, and result of human tumors^9,10^. DNA methylation serves as a significant epigenetic modification of cancer cells, which may be the main mechanisms of the inactivation of cancer-associated suppressor genes in the process of tumorigenesis^11^. Various studies suggested that DNA methylation was closely related to the development, invasion, and metastasis of carcinoma, and may act as a hallmark of prognosis^12,13^. For example, Guo et al. revealed a five-DNA methylation signature as an effective prognostic hallmark in patients with ovarian serous cystadenocarcinoma^14^. Li et al. suggested that a four-DNA methylation signature served as a robust prognostic biomarker for survival of patients with gastric cancer^15^. In addition, a previous study reported that methylation was more likely to occur for DNA in tumor cells than that in normal cells^16^. Nielsen et al. indicated that aberrant DNA methylation was a relatively stable as well as potentially reversible therapeutic signal^17^. Therefore, DNA methylation has great potential as biomarker of prognosis for cancer patients. However, few studies have investigated the value of DNA methylation for prognostic prediction in patients with stage I–II LUAD. The identification of novel prognostic DNA methylation hallmark for stage I–II LUAD patients was highly desired.

We analyzed the genome-wide methylation map of stage I–II LUAD patients in TCGA database to investigate a DNA methylation-based predicator and a DNA methylation-associated nomogram. The Kaplan–Meier survival curve and ROC analyses were employed to test the robustness of the 11-DNA methylation signature and the results suggested a good value of our nomogram.

## Results

### Clinical characteristics of the study populations

**Figure 1:**
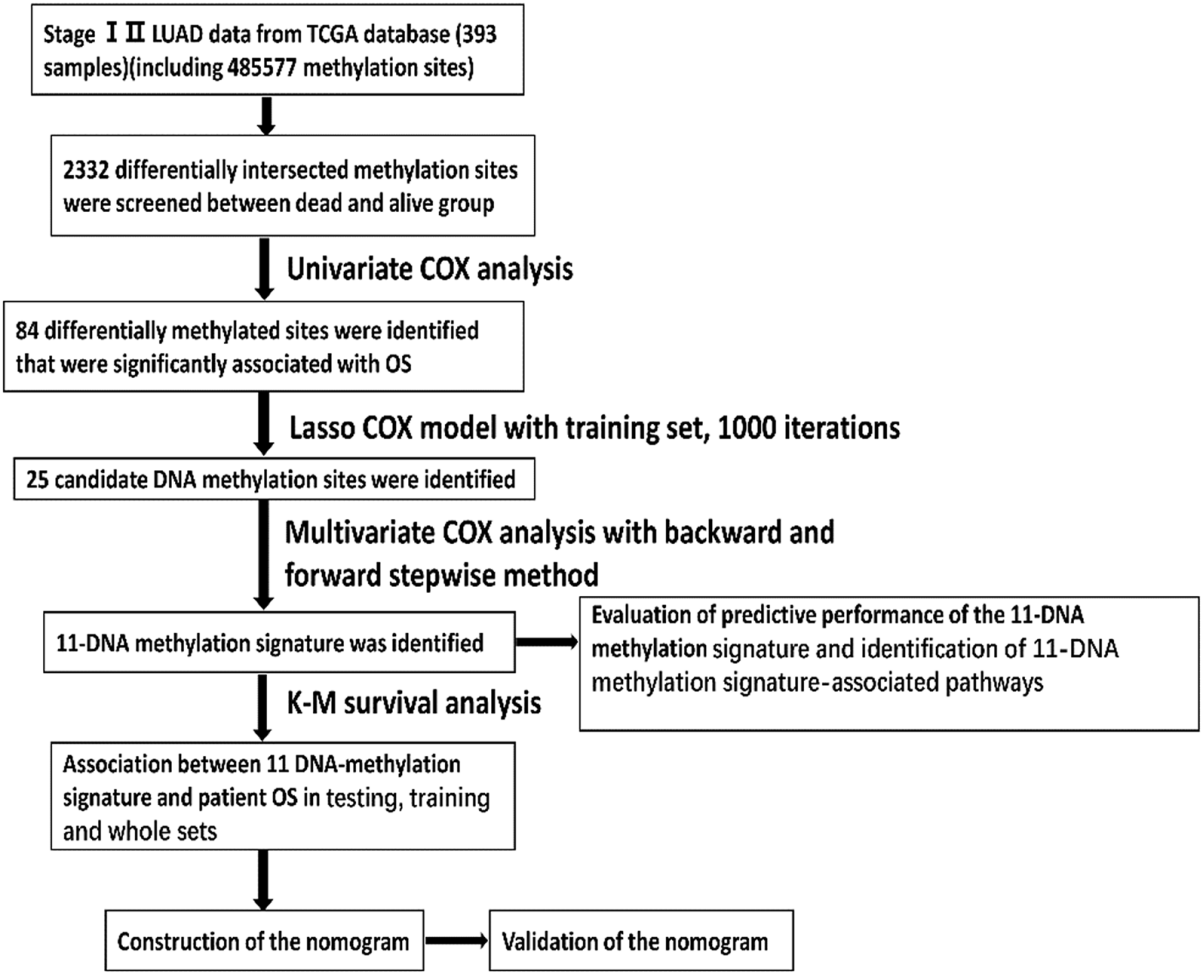
Workflow of model generation and subject enrolment.

A total of 393 stage I–II LUAD samples were enrolled in the present study. The demographics and clinical features of the included patents are summarized in Table 1. Workflow of model generation and subject enrolment was summarized in Fig. 1.

**Table 1:** Clinical features of enrolled samples.

Characteristics	Total (n = 393)	Training dataset (n = 276)	Testing dataset (n = 117)
**Sex**			
Female	212 (53.94)	144 (52.17)	68 (58.12)
Male	181 (46.06)	132 (47.83)	49 (41.88)
**Age**			
≤ 65	195 (49.62%)	140 (50.72%)	55 (47.01%)
> 65	189 (48.09%)	130 (47.1%)	59 (50.43%)
Not available	9 (2.29%)	6 (2.17%)	3 (2.56%)
**Stage**			
Stage I	275 (69.97%)	192 (69.57%)	83 (70.94%)
Stage II	118 (30.03%)	84 (30.43%)	34 (29.06%)
**M**			
M0	263 (66.92%)	194 (70.29%)	69 (58.97%)
MX	125 (31.81%)	78 (28.26%)	47 (40.17%)
Not available	5 (1.27%)	4 (1.45%)	1 (0.85%)
**T**			
T1	154 (39.19%)	107 (38.77%)	47 (40.17%)
T2	209 (53.18%)	151 (54.71%)	58(49.57%)
T3	30 (7.63%)	18 (6.52%)	12 (10.26%)
**N**			
N0	306 (77.86%)	208 (75.36%)	98 (83.76%)
N1	75 (19.08%)	58 (21.01%)	17 (14.53%)
NX	12 (3.05%)	10 (3.62%)	2 (1.71%)
**Location**			
Central lung	45 (11.45%)	33 (11.96%)	12 (10.26%)
Peripheral lung	84 (21.37%)	63 (22.83%)	21 (17.95%)
Not available	202 (51.4%)	134 (48.55%)	68 (58.12%)
Unknown	62 (15.78%)	46 (16.67%)	16 (13.68%)
**Neoadjuvant treatment**			
No	391 (99.49%)	274 (99.28%)	117 (100%)
Yes	2 (0.51%)	2 (0.72%)	
**Site**			
L-Lower	64 (16.28%)	47 (17.03%)	17 (14.53%)
L-Upper	87 (22.14%)	59 (21.38%)	28 (23.93%)
R-Lower	61 (15.52%)	44 (15.94%)	17 (14.53%)
R-Middle	13 (3.31%)	8 (2.9%)	5 (4.27%)
R-Upper	160 (40.71%)	114 (41.3%)	46 (39.32%)
Not available	8 (2.04%)	4 (1.45%)	4 (3.42%)
**Residual tumor**			
R0	252 (64.12%)	175 (63.41%)	77 (65.81%)
R1	4 (1.02%)	3 (1.09%)	1 (0.85%)
Not available	137 (34.86%)	98 (35.51%)	39 (33.33%)
**Race**			
Asian	4 (1.02%)	3 (1.09%	1 (0.85%)
Black or African American	44 (11.2%)	31 (11.23%)	13 (11.11%)
White	315 (80.15%)	221 (80.07%)	94 (80.34%)
Not available	30 (7.63%)	19 (6.88%)	11 (9.40%)

### Methylation markers associated with OS of stage I–II LUAD patients

**Figure 2:**
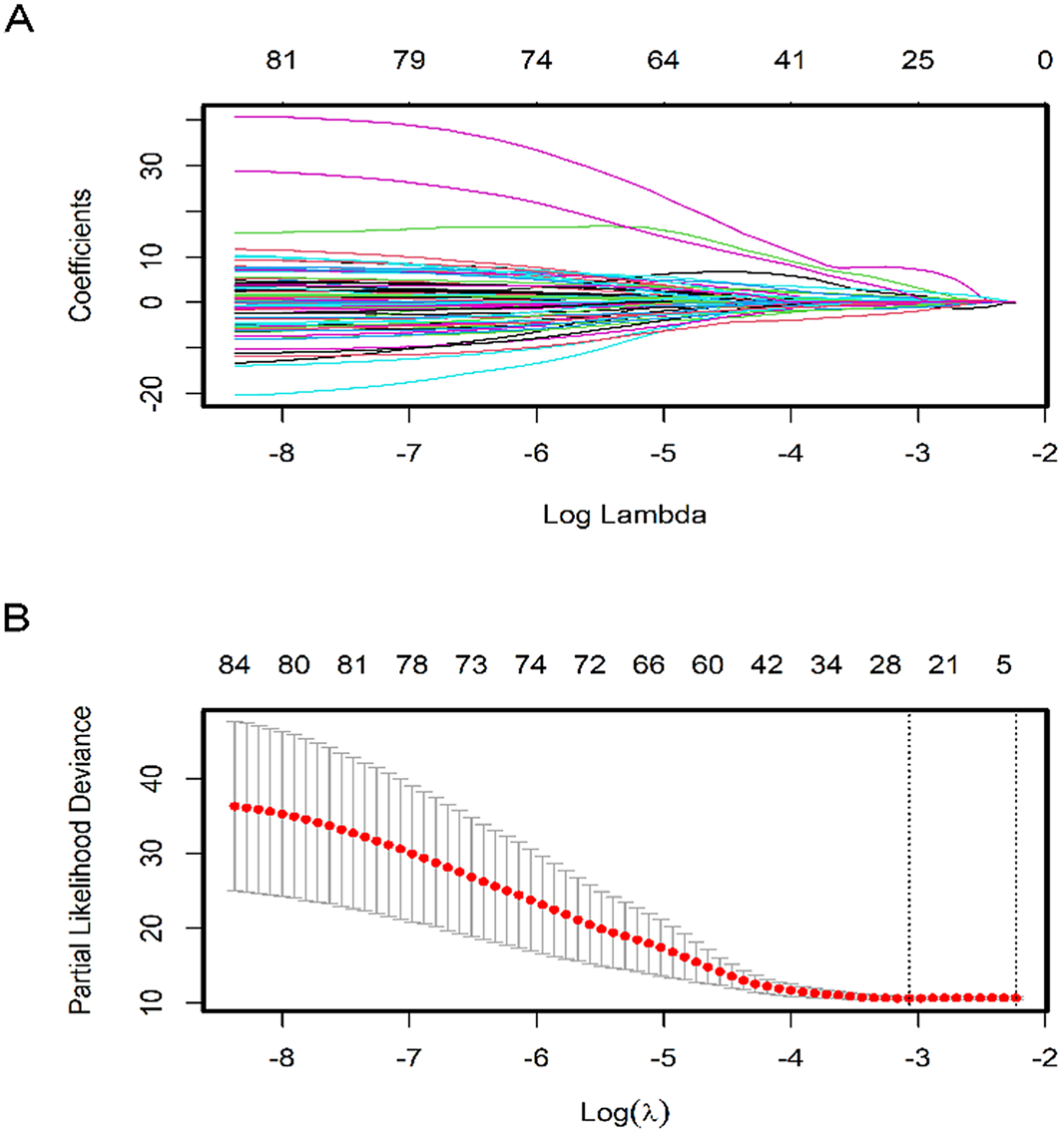
Candidate methylation sites selection on the basis of the LASSO Cox regression model. (**A**) LASSO Cox regression with L1 regularization. Tenfold cross-validation for tuning parameter selection in the LASSO model via minimum criteria (the 1-SE criteria). (**B**) The LASSO Cox regression model was employed to determine the most robust hallmarks. LASSO coefficient profiles of the 84 methylation sites. A coefficient profile plot was created against log (lambda) sequence. Vertical line was used at the value selected by using tenfold cross-validation, where optimal lambda resulted in 25 non-zero coefficients.

**Figure 3:**
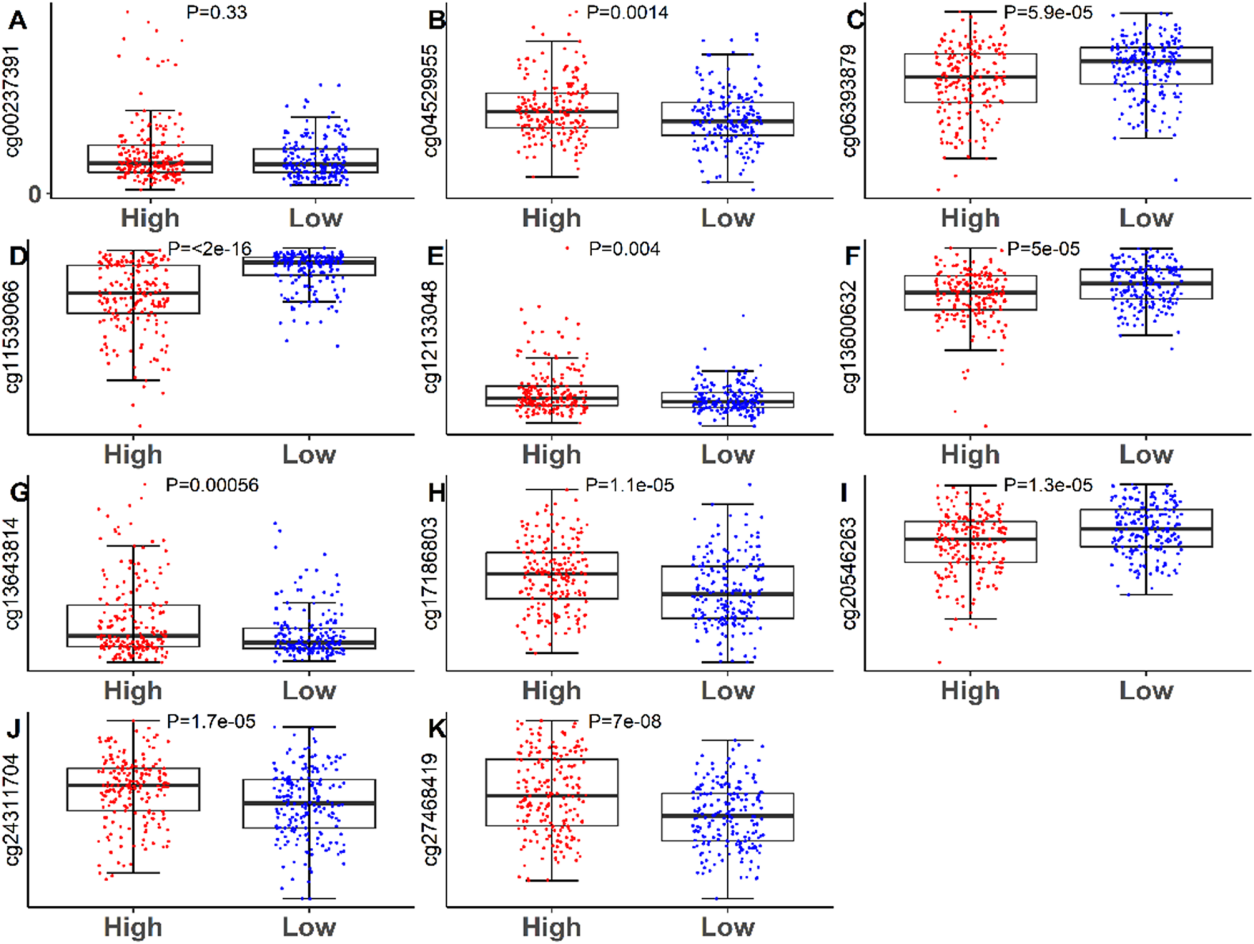
Boxplots of methylation β values against risk group in the entire TCGA dataset. “High Risk” and “Low Risk” represent the high-risk and low-risk samples, respectively. The median risk score was applied as a cutoff. Vertical coordinates represent the β-value of 11-DNA methylation sites respectively. Mann–Whitney U test was employed to assess the diferences between the high-risk score and low-risk score groups.

We identified 2332 differentially expressed methylation sites between dead and alive cohorts that were adopted for univariate Cox proportional hazard regression analysis. Consequently, 84 DNA methylation sites were revealed to be closely involved in stage I–II LUAD patients’ OS (P < 0.05). Following this, the identified 84 DNA methylation sites were used for LASSO Cox regression model (Supplementary Table S1) and 25 methylation sites were determined as the candidate methylation sites (P < 0.01) (Fig. 2A,B) that were then used for multivariate Cox proportional hazard regression. Of these, 11 methylation sites were screened as optimal model sites for the prognostic evaluation of patients with stage I–II LUAD based on the multivariate Cox regression analysis. The risk scoring formula was calculated as follows: Risk score = 1.31688*cg00237391 + 8.85449*cg04529955 − 3.3827*cg06393879 − 3.34536*cg11539066 + 2.62432*cg12133048 − 6.18862*cg13600632 + 1.50514*cg13643814 + 2.3872*cg17186803 − 3.74192*cg20546263 + 2.57421*cg24311704 + 1.19749*cg27468419. The scores showed strong associations of poor prognosis with hypermethylation of cg00237391, cg04529955, cg12133048, cg13643814, cg17186803, cg24311704, cg27468419 and the hypomethylation of cg06393879, cg11539066, cg13600632 and cg20546263 sites (Fig. 3).

### Association between the 11-DNA methylation signature and stage I–II LUAD patients’ OS

**Figure 4:**
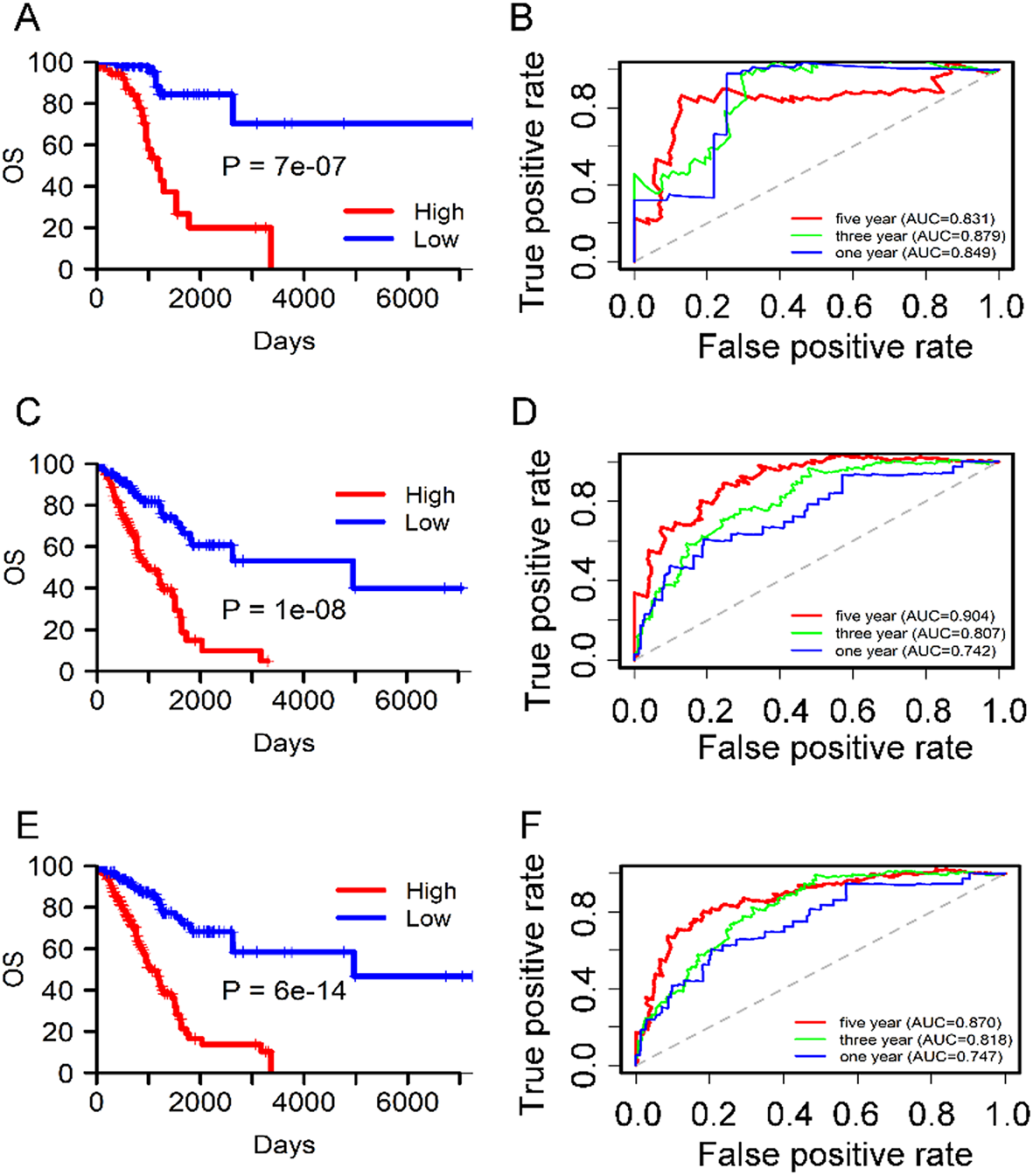
Kaplan–Meier and ROC analysis of patients with stage I–II LUAD in in training, validation and whole datasets. (**A**, **C**, **E**) Kaplan–Meier analysis for stage I–II LUAD patients between the low-risk and high-risk. The x-axis is follow-up time, the y-axis is OS. The log-rank test indicated the higher risk scores were significantly correlated with worse OS (P < 0.05). (**B**, **D**, **F) **1-, 3-, 5-year ROC curves of the 11-DNA methylation signature. Blue line, green line and red line represent 1-, 3-, 5-year ROC curves respectively.

The prognostic value of the selected 11-DNA methylation hallmark was assessed on the basis of Kaplan–Meier survival analyses in the training, validation and the all-cohort datasets. The median risk scores were employed as cutoff value to divide the patients into low and high risk groups. As exhibited in Fig. 4A, OS was significantly longer in the low-risk cohort in comprison to the high-risk cohort in the training dataset (P = 7e − 07). A similar result was found in the validation dataset (P = 1e − 08) (Fig. 4C) and in the all-cohort dataset (P = 6e − 14) (Fig. 4E). The results implied a great robustness of the predicator as prognostic indicator in patients with stage I–II LUAD.

### Evaluation of the predictive ability of the 11-DNA methylation biomarker by ROC analysis

We performed ROC analysis to further evaluate the value of the 11-DNA methylation biomarker. The AUC of the 11-DNA methylation hallmark at 1, 3, 5 years in training set were 0.849, 0.879, 0.831, respectively (Fig. 4B). A good value was also found in validation set (0.742, 0.807, 0.904, respectively) (Fig. 4D) and the whole validation set (0.747, 0.818, 0.870, respectively) (Fig. 4F), the results showed that the 11-DNA methylation biomarker had a significant capacity for predicting OS of stage I–II LUAD patients.

**Figure 5:**
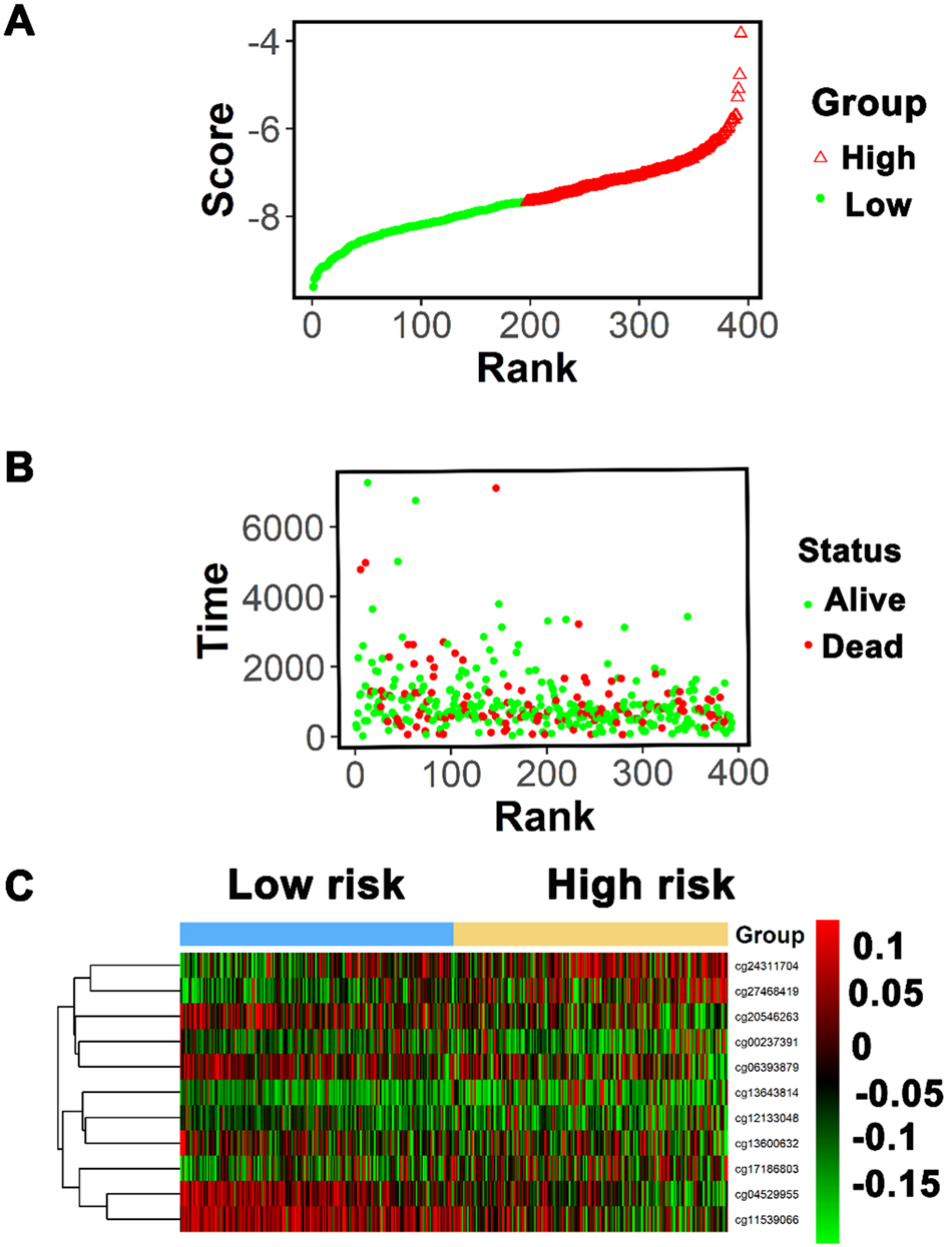
Methylation risk score analysis of 393 stage I–II LUAD cases in the entire TCGA dataset. (**A**) Methylation risk score distribution against the rank of risk score. The red triangle represented the high-risk samples, the green ball represented the low-risk samples. (**B**) Survival status of stage I–II LUAD patients against the rank of risk score. The green ball referred to alive samples, the red ball referred to the dead samples. (**C**) Heatmap showed 11 methylation sites profiles in low- and high-risk groups. Each row of the heat map represented a profile of a methylation site.

Then, the patients enrolled in this study were ranked based on their risk scores (Fig. 5A), and the dotplot was drawn in based on their recurrence status (Fig. 5B). The outcomes showed that the low-risk cohort had a longer OS than that in the high-risk cohort. Heatmap of 11 methylation sites distribution on the basis of risk score was observed in Fig. 5C, which had a similar result to our previous boxplot. Finally, we performed subgroup analysis by using several clinicopathological factors (site, age, stage, gender and smoking history). A good predictive ability of the 11-DNA methylation biomarker was verified in most sub-group (Supplementary Figs. S1, Figs. S2, Figs. S3, Figs. S4 and Figs. S5).

### Implementation of the 11-DNA methylation biomarker-related biological pathways

**Figure 6:**
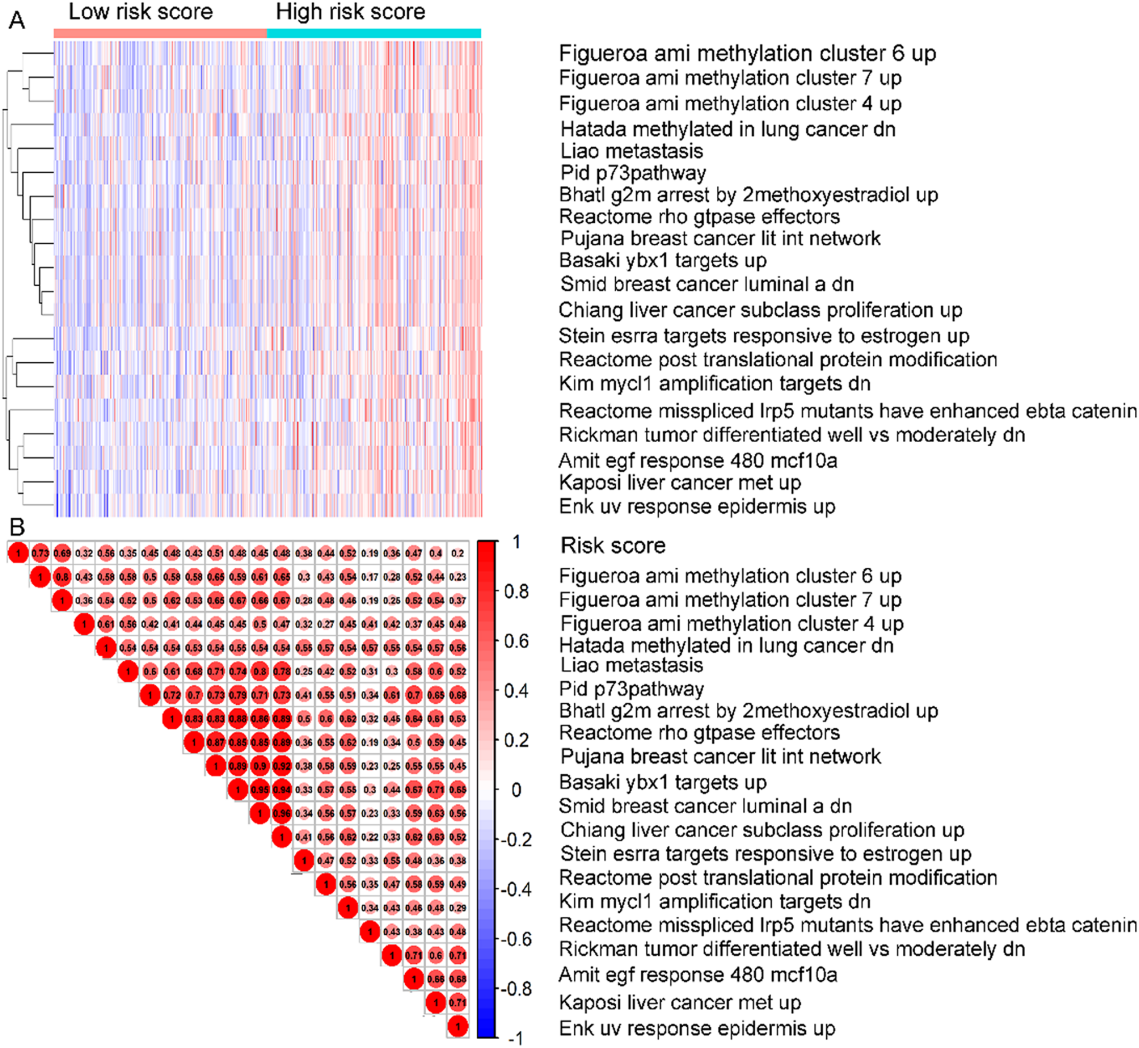
Exploration of the 11 DNA methylation signature-based biological pathways. (**A**) Heatmap of top 20 enriched pathways associated with high risk group. Each row of the heat map represented a pathway and each column represented a stage I–II LUAD sample. (**B**) Correlation graph between risk scores and top 20 pathways. Each red ball represents a pathway, and each transverse line represents one sample. The score characteristic of each sample was shown in the graph.

Single-sample Gene Sets Enrichment Analysis (ssGSEA) was carried out in accordance to TCGA LUAD mRNA dataset by employing GSVA package^18^ for exploring the 11-DNA methylation signature-based signaling pathways. The median score was employed as the cutoff to divide the patients into low and high risk groups. Top 20 pathways that were more activated in the high-risk samples than that in low-risk samples were exhibited in Fig. 6A (Supplementary Table S2). The exact Pearson correlations between enriched pathways and risk score was presented in Fig. 6B.

### Nomogram development

**Figure 7:**
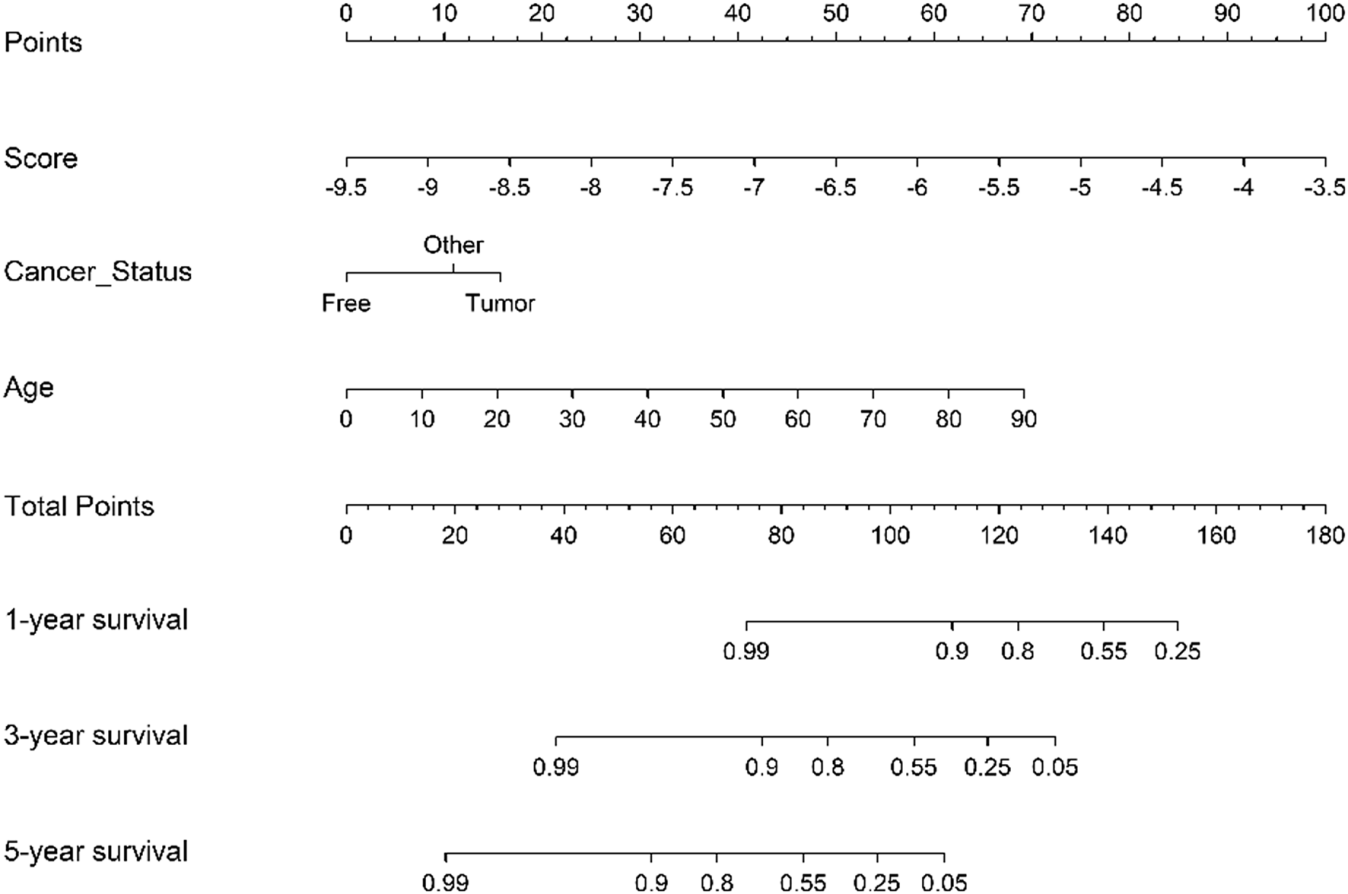
In order to quantify the risk assessment and survival probability for individual stage I–II LUAD patients, a nomogram was developed in the entire TCGA dataset according to the 11 DNA methylation signature-based risk score, age and cancer status.

**Figure 8:**
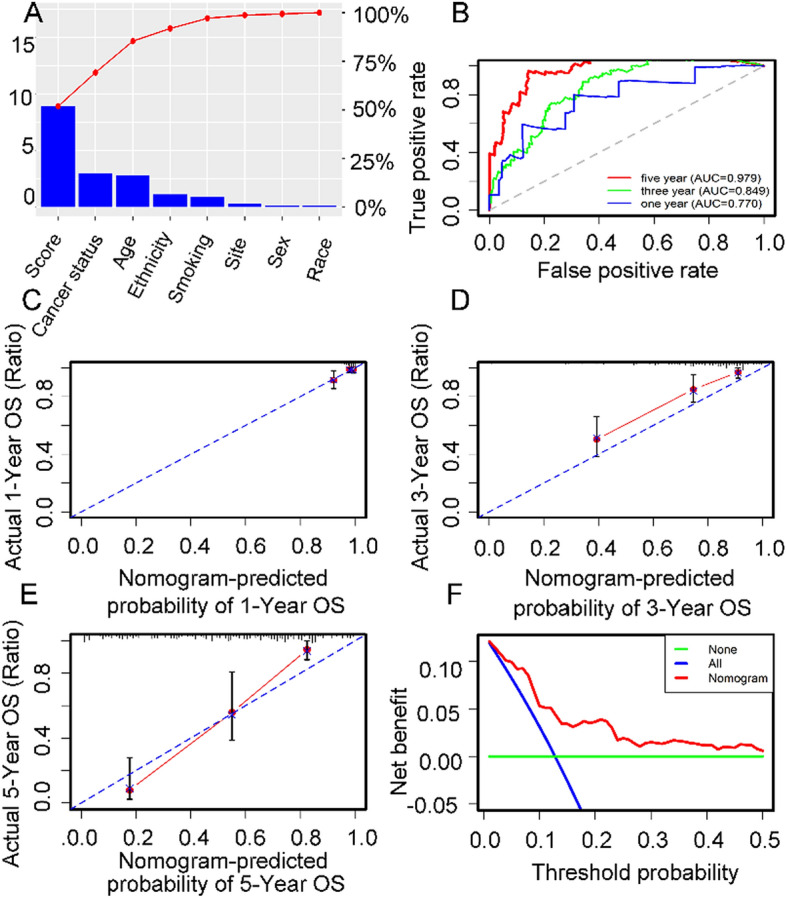
Analysis of the 11-DNA methylation biomarker-related nomogram in the entire TCGA dataset. (**A**) The horizontal axis stood for clinical factors, the vertical axis stood for the percentage of importance. (**B**) 1-, 3-, 5-year ROC curves for the 11-DNA methylation biomarker-related nomogram. (**C**–**E**) referred to the 1-, 3-, 5-year nomogram calibration curves, respectively. (**F**) The DCA for the nomogram. The net benefit was plotted versus the threshold probability. The red line represented the nomogram. The blue line represented the treat-all and the green line represented the treat-none.

To assess whether the 11-DNA methylation biomarker was an independent predictor for OS of stage I–II LUAD patients, we carried out univariate and multivariate Cox model via risk score and a few clinicopathological factors. Hazard ratios (HRs) showed that the 11-DNA methylation hallmark was significantly involved in the OS of stage I–II LUAD patients (P < 0.01, HR 2.60, 95% CI 1.91–3.53) (Table 2), which implied that the 11-DNA methylation hallmark was an independent prognosis classifier. To further predict OS of stage I–II LUAD patients via a quantitative strategy, we developed a nomogram (Fig. 7) based on risk score, age and cancer status. The significance between risk score and other clinical factors was found in Fig. 8A. The value of the nomogram was measured based on C-index (0.787, 95% CI 0.751–0.832), AUC (1, 3, 5-year: 0.770, 0.849, 0.979) (Fig. 8B) and calibration plot (Fig. 8C–E), the results suggested a good value of our nomogram. Besides, calibration plot and decision curve analysis (DCA) (A novel method for evaluating prediction models) suggested that the nomogram had crucial clinical appliance potential for the prognostication of stage I–II LUAD patients’ OS than that in treat all or treat none cohort. Net benefit was proved for stage I–II LUAD patients in 3-year recurrent risks (Fig. 8F). The result indicated that our methylomics-associated nomogram had a strong robustness and may have clinical application potential.

**Table 2:** Univariate Cox regression analysis and multivariate Cox regression analysis outcome on the basis of risk score and other clinical factors.

ID	Univariate Cox analysis				Multivariate Cox analysis			
	HR	HR.95L	HR.95H	P value	HR	HR.95L	HR.95H	P value
Score	3.0302306	2.296882692	3.99772146	4.44E − 15	2.59759935	1.9090689	3.5344572	1.24E − 09
Cancer status	0.4924994	0.365731098	0.663207593	3.09E − 06	0.60389188	0.4468942	0.8160442	0.001026
Age	1.0486502	1.022854165	1.075096827	0.0001854	1.04561408	1.0169743	1.0750604	0.001645
Ethnicity	0.4618099	0.252547796	0.844467451	0.0121097	0.56260564	0.2980827	1.0618701	0.075942
Smoking number	1.0121688	1.00413782	1.020263986	0.002921	1.00730659	0.9979011	1.0168007	0.128261
Location	0.6257271	0.411043888	0.95253669	0.0287594	0.87308145	0.572785	1.3308156	0.527977
Sex	0.6849284	0.412997794	1.135906531	0.1425824	0.92573233	0.5377101	1.593759	0.780697
Race	1.6990724	1.099136566	2.626467912	0.0170644	1.06966614	0.660713	1.7317438	0.784103
Residual tumor	0.7152782	0.40245641	1.271250417	0.2534502				
History	1.4308373	0.76004129	2.693663511	0.2670376				
N	0.3970412	0.054727983	2.880458938	0.3609272				
M	0.772249	0.442725232	1.347039772	0.3625725				
Smoking history	0.9727666	0.769862071	1.229148509	0.8170547				
T	1.0463717	0.637579911	1.717265142	0.8576759				
Site	1.0012003	0.887959798	1.128882291	0.9843716

### Comparison of our nomogram with several known prognostic hallmarks

**Figure 9:**
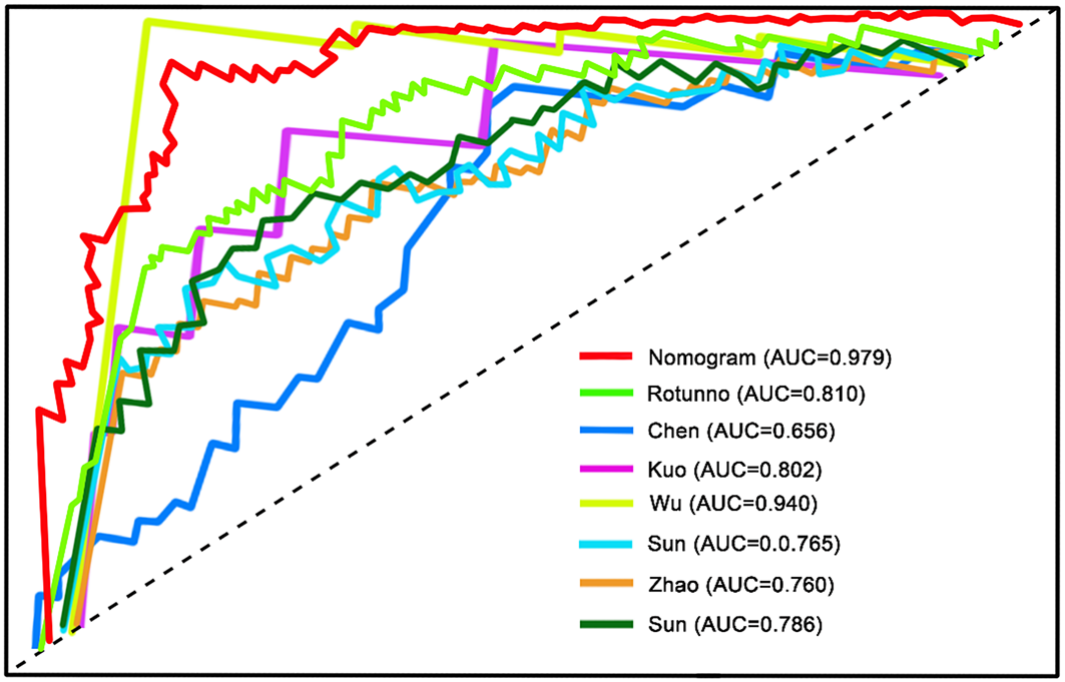
ROC curves illustrated the effectiveness of the methylation-related nomogram and a few known biomarkers in predicting the prognosis of stage I–II LUAD patients. The AUC in Rotunno et al. is 0.810, in Chen et al. (0.656), in Kuo et al. (0.802), in Wu et al. (0.940), in Sun et al. (0.765), in Zhao et al. (0.760), in Sun et al. (0.786).

We compared the nomogram of this study with other known prognostic biomarkers to confirm whether the nomogram hallmark had the advantage of stable and great performance. The biomarkers for predicting early stage LUAD patients' OS were employed for the comparison^19–25^. As indicated in Fig. 9, the result manifested that the nomogram outperformed a few known prognostic hallmarks. The AUC of the nomogram at 5 years was 0.979, which was obviously larger than that in other biomarkers, suggestive of a greater value for the nomogram in comparison to other signatures in predicting LUAD patients' prognosis.

## Discussion

Despite advances in diagnosis and therapy of NSCLC over the past few decades, the 5-year survival rate is still poor^3^. Thus, it is urgently needed to identify potential hallmarks correlated with the prognosis of NSCLC and develop optimum targeted therapy. With the deepening study of epigenetics, increasing studies have suggested that DNA methylation is crucial to gene regulation and acts as early events of a few tumors. DNA methylation serves as one of the earliest detectable neoplastic alterations, which give it a significant superiority as carcinoma diagnosis and prognosis hallmarks^26–28^. Various studies indicated that DNA methylation could function as predicators for cancer patients. For instance, Zheng et al. revealed a prognostic 11-DNA methylation biomarker for lung squamous cell carcinoma^29^. Peng et al. identified a DNA methylation signature to improve survival prediction of gastric cancer^30^.

In the present study, TCGA database were applied to analyze the methylation of stage I–II LUAD. We identified a signature which contained 11 methylation sites (cg00237391 (DEF6: 1stExon), cg04529955 (SLC10A7: Body), cg06393879 (MYT1L: Body), cg11539066 (MIR596: TSS1500), cg12133048, cg13600632 (FAM125B: Body), cg13643814 (CHRNA7: TSS1500), cg17186803 (CN4B: Body), cg20546263 (C3orf33: TSS1500), cg24311704 (MUC21: 1stExon) and cg27468419 (SDR16C6: TSS1500)) and corresponded to ten genes (DEF6, SLC10A7, MYT1L, MIR596, FAM125B, CHRNA7, SCN4B, C3orf33, MUC21, SDR16C6) by differential methylation analysis, Kaplan–Meier survival analysis, ROC analysis, and Cox regression analysis. Interestingly, previous studies have indicated that most of these ten genes were related to cancer, respectively. For example, Liew et al. suggested that DEF6 expression in ovarian carcinoma was associated with poor survival of patients^31^. Liu et al. indicated that SLC10A7 was involved in prognosis-associated alternative splicing events of cancer^32^. Zhang et al. revealed the clinical significance of MYT1L gene polymorphisms in Chinese patients with gastric cancer^33^. Liu et al. reported that miR-596 could modulate melanoma growth via regulating cell survival and death^34^. Xiang et al. found that CHRNA7 inhibited cell invasion and metastasis of LoVo human colorectal cancer cells by PI3K/Akt signaling^35^. Dai et al. revealed that miR-424-5p promoted the proliferation and metastasis of colorectal cancer via directly targeting SCN4B^36^.Yuan et al. identified a novel splice variant of AC3-33 (C3orf33) in breast cancer^37^. Kai et al. discovered that mucin 21 was a novel, negative immunohistochemical marker for epithelioid mesothelioma for its differentiation from LUAD^38^. The result showed that 8 of these 10 genes were involved in cancer. We speculated the above 10 genes may be related to OS of LUAD patients. In addition, the result of Fig. 3 suggested that hypermethylation of cg00237391, cg04529955, cg12133048, cg13643814, cg17186803, cg24311704, cg27468419 sites was involved in poor OS and the hypomethylation of cg06393879, cg11539066, cg13600632 and cg20546263 sites was associated with a shorter OS. The prognosis predictive ability of a single methylation site was limited while the combination of multiple methylation sites had a stronger robustness for predicting OS of LUAD patients, which was further verified by Fig. 4B,D,F.

To better explore whether different clinical characteristics have different effects on the 11-DNA methylation signature, we divided the patients based on site, age, stage, gender and smoking history and used the subgroup analysis to detect whether there were differences between ROC curves of different clinical characteristics cohorts. An 11-DNA methylation signature was determined in the present study. The result indicated that the biomarker had a high diagnostic ability for prognosis of stage I–II LUAD patients. We hope that it can assist monitor stage I–II LUAD patients’ OS and also offer some assistance for the in-depth study of stage I–II LUAD. In addition, a few significant superiorities were required to be elaborated in the study. We developed a nomogram based on risk score, age and cancer status to predict 3- and 5-year stage I–II LUAD patients’ OS. The result suggested that AUC at 1, 3, 5 years entire TCGA dataset were 0.770, 0.849, 0.979, respectively, indicating good value of the 11-DNA methylation biomarker in the clinical application, which made our study more valuable. On the other hand, we performed LASSO Cox regression analysis to explore the candidate methylation sites significantly involved in stage I–II LUAD patients’ OS, which can filter the variables between univariate and multivariate Cox analysis. That is to say, the application of LASSO Cox regression model can elevate the predictive value of the 11-DNA methylation biomarker.

Following that, a comparison of the nomogram with other known prognostic predicators exhibited that our nomogram had apparently higher value in the result prediction of LUAD. In addition, the AUC of the nomogram was larger than the 11-DNA methylation biomarker in this study, demonstrating that the combination of the risk score with clinical variables was more significant in comparison to the methylation-correlated signature alone in the prediction of LUAD patients' prognosis. In addition, based on our nomogram, clinicians will be able combine clinical and molecular factors to determine an individualized probability of recurrence for patients with stage I–II LUAD, which represents a major advance in the field of personalized medicine for pulmonary oncology, suggesting that this tool could help clinicians overcome one of the most challenging limitations we face when treating patients with stage I–II LUAD.

Whereas, there were a few limitations that needed to be emphasized in this study. Firstly, an independent external validation set was required to improve the predictive accuracy of DNA methylation biomarker in our study. Secondly, it takes a relatively long time for this predictive marker to be used clinically. Thirdly, our study was a retrospective study from TCGA database, which might produce some amount of selection bias. In addition, the samples of our study contained a mixture of methylomes in a tumor-lymphocytes/subsets, macrophage/subsets, fibroblasts, as well as tumor cells, which may make reproducibility challenge. Furthermore, Illumina's 450 K Platform chip measured 450,000 methylation sites, which was less than 1% of all methylation sites, and this may yield some bias in our study.

## Conclusions

In this study, we successfully developed a new DNA methylation prognostic predicator. In addition, a nomogram was developed based on methylation risk score, age and cancer status by a comprehensive analysis of bioinformatics methods, which could be used for the prediction of stage I–II LUAD patients’ OS.

## Materials and methods

### DNA methylation data of stage I–II LUAD patients

DNA methylation data of patients with stage I–II LUAD from TCGA database that was processed using the Illumina HumanMethylation450 BeadChip (Illumina Inc., CA, USA) and the associated clinical information was retrieved from TCGA database by R TCGAbiolinks package^39^, notably, Illumina's 450 K Platform chip analyzed 450,000 methylation sites, which was less than 1% of all methylation sites, and this may yield some bias in our study. The DNA methylation were evaluated via β-values and calculated as the proportion of M and M + U + 100, in which M represented the signal from methylated beads, and U referred to the signal from unmethylated beads targeting CpG site. Data for a total of 393 stage I–II LUAD patients containing 485,577 DNA methylation sites were enrolled after removing patients for whom clinical survival information was not available. We analyzed the correlation between DNA methylation levels and the related OS of patients with stage I–II LUAD. We randomly divided the total stage I–II LUAD patients into two parts: training group (70%) and testing group (30%). The training group was used for model construction and the testing group for assessing the value of the model. LASSO is a critical regularization in many various analysis approaches. Here, LASSO regression model was used to screen core methylation sites involved in stage I–II LUAD patients’ OS. LASSO COX regression analysis was conducted by using a publicly available R package ‘glmnet’^40^ for 1000 iterations.

### Data processing, normalization, and determination of differentially expressed methylation sites

The preprocessing of the raw data was performed to analyze the prognosis prediction classifier. The methylation site whose beta value was not available (NA) in any sample was deleted. Following this, we performed the normalization of the data by ‘betaqn’ function of wateRmelon package^41^. Moreover, the whole samples were divided into dead and alive groups based on recurrent status. The normalized beta was transformed to M value with the formulation: M = log(β/(1 − β)). M value was employed for the elimination of the bias caused by different probes. Finally, M value was exploited for the determination of the differentially expressed methylation sites between recurrent cluster and no recurrent cluster with ‘dmpFinder’ function of minfi package^42^.

### Identification of methylomics-based signature

First of all, Univariate Cox regression analysis was conducted to screen the methylation sites associated with the OS of stage I–II LUAD patients. Then, the LASSO Cox regression analysis was performed via the screened methylation sites for the selection of the candidate methylation sites correlated with OS of stage I–II LUAD patients. Following this, the candidate methylation sites were analyzed by multivariate Cox regression analysis to determine the independent prognostic hallmark for OS of stage I–II LUAD patients. Eventually, an 11-DNA methylation signature was determined for the prediction of OS stage I–II LUAD patients. Risk score models were developed on the basis of the 11-DNA methylation signature to calculate the risk score of each sample. The median risk score was set as the cutoff point. Patients with stage I–II LUAD were categorized into “high-risk” or “low-risk” cohorts based on a high and low score, respectively. Log-rank testing of the Kaplan–Meier curve was performed via the “survival” package^43^ to assess the difference in OS of the two cohorts. ROC analysis was performed via the ‘survivalROC’ package^44^ and the area under the ROC curve (AUC) was employed to evaluate the predictive performance of the hallmark. The greater the AUC value of a hallmark, the better the predictive capacity of the marker. Unless otherwise noted, all curves were plotted using R (version 4.0.2$2020).

### Gene set variation analysis (GSVA)

To reveal the established biomarker-associated signaling pathways, we used a GSVA package^18^ to evaluate DNA methylation risk score and enriched pathways activity conditions. The median risk score was set as the cutoff point to divide the patients into “high-risk” or “low-risk” cohorts. The exact pearson was drawn to analyze the correlations between enriched pathways and risk score. Significance was set as P < 0.05.

### Construction of the nomogram

To improve the predictive value of established predicator for stage I–II LUAD patients’ OS, a nomogram was built via the ‘rms’ R package^45^. The univariate and multivariate Cox proportional hazard analysis were conducted on the basis of the methylation risk score and other clinical factors. Cox proportional hazard models was adopted to measure hazard ratios (HR) and corresponding 95% confidence interval (CI). We used the factors (P ≤ 0.05) from the multivariate Cox proportional hazard analysis to construct the nomogram. The nomogram was assessed based on C-index, ROC, DCA. The calibrate curve described the outcome of the nomogram, and the 45° line suggested the best prediction.

### Ethics approval and consent to participate

Data obtained from the TCGA open-access database was collected from tumors of patients who provided informed consent based on the guidelines from the TCGA Ethics, Law and Policy Group.

### Consent for publication

All patients included in the TCGA public domain database consented for publication as detailed in [https://cancergenome.nih.gov/abouttcga/policies/informedconsent].

## Supplementary Information

Supplementary Figure S1.

Supplementary Figure S2.

Supplementary Figure S3.

Supplementary Figure S4.

Supplementary Figure S5.

Supplementary Table S1.

Supplementary Table S2.

## Data Availability

All data generated or analyzed during this study are included in this published article (and its “Supplementary Information” files).

## References

[1] Khalil S, Hatch L, Price CR (2020). Addressing breast cancer screening disparities among uninsured and insured patients: A student-run free clinic initiative. J. Community Health.

[2] Molina JR, Yang P, Cassivi SD (2008). Non-small cell lung cancer: Epidemiology, risk factors, treatment, and survivorship. Mayo Clin. Proc..

[3] Consonni D, Pierobon M, Gail MH (2015). Lung cancer prognosis before and after recurrence in a population-based setting. J. Natl. Cancer Inst..

[4] Akagi I, Okayama H, Schetter AJ (2013). Combination of protein coding and noncoding gene expression as a robust prognostic classifier in stage I lung adenocarcinoma. Can. Res..

[5] Wang C, Jiang Y, Lei Q (2019). Potential diagnostic and prognostic biomarkers of circular RNAs for Lung cancer in China. Biomed. Res. Int..

[6] Ning Y, Liu W, Guan X (2019). CPSF3 is a promising prognostic biomarker and predicts recurrence of non-small cell lung cancer. Oncol. Lett..

[7] Liu HY, Lu SR, Guo ZH (2020). lncRNA SLC16A1-AS1 as a novel prognostic biomarker in non-small cell lung cancer. J. Investig. Med. Off. Publ. Am. Federation Clin. Res..

[8] Zhang, S. *et al*. Identification six metabolic genes as potential biomarkers for lung adenocarcinoma. *J. Comput. Biol.* **27**(10), 1532–1543. 10.1089/cmb.2019.0454. (2020). **Epub 16 Apr 2020.** 10.1089/cmb.2019.0454 32298601

[9] Cai L, Bai H, Duan J (2019). Epigenetic alterations are associated with tumor mutation burden in non-small cell lung cancer. J. Immunother. Cancer.

[10] Azmi, A.S. *et al*. DNA-methylation-caused downregulation of miR-30 contributes to the high expression of X. *Cancers (Basel)*. **11**(8), 1101. 10.3390/cancers11081101 (2019). 10.3390/cancers11081101 PMC6721494 31382411

[11] Ghavifekr Fakhr M, Farshdousti Hagh M, Shanehbandi D (2013). DNA methylation pattern as important epigenetic criterion in cancer. Genet. Res. Int..

[12] Klutstein M, Nejman D, Greenfield R (2016). DNA methylation in cancer and aging. Can. Res..

[13] Molnár KB (2020). Analysis of DNA methylation alterations in cellfree DNA fraction during colorectal cancer development. Magy. Onkol..

[14] Guo W, Zhu L, Yu M (2018). A five-DNA methylation signature act as a novel prognostic biomarker in patients with ovarian serous cystadenocarcinoma. Clin. Epigenet..

[15] Li C, Zheng Y, Pu K (2020). A four-DNA methylation signature as a novel prognostic biomarker for survival of patients with gastric cancer. Cancer Cell Int..

[16] Aran D, Hellman A (2013). DNA methylation of transcriptional enhancers and cancer predisposition. Cell.

[17] Nielsen SN, Grell K, Nersting J (2017). DNA-thioguanine nucleotide concentration and relapse-free survival during maintenance therapy of childhood acute lymphoblastic leukaemia (NOPHO ALL2008): A prospective substudy of a phase 3 trial. Lancet Oncol..

[18] Hänzelmann S, Castelo R, Guinney J (2013). GSVA: Gene set variation analysis for microarray and RNA-seq data. BMC Bioinform..

[19] Chen M, Liu B, Xiao J (2017). A novel seven-long non-coding RNA signature predicts survival in early stage lung adenocarcinoma. Oncotarget.

[20] Sun Y, Hou L, Yang Y (2016). Two-gene signature improves the discriminatory power of IASLC/ATS/ERS classification to predict the survival of patients with early-stage lung adenocarcinoma. Onco. Targets. Ther..

[21] Sun J, Zhao T, Zhao D (2020). Development and validation of a hypoxia-related gene signature to predict overall survival in early-stage lung adenocarcinoma patients. Therap. Adv. Med. Oncol..

[22] Zhao Z, Zhao D, Xia J (2020). Immunoscore predicts survival in early-stage lung adenocarcinoma patients. Front. Oncol..

[23] Wu P, Zheng Y, Wang Y (2020). Development and validation of a robust immune-related prognostic signature in early-stage lung adenocarcinoma. J. Transl. Med..

[24] Kuo IY, Jen J, Hsu LH (2016). A prognostic predictor panel with DNA methylation biomarkers for early-stage lung adenocarcinoma in Asian and Caucasian populations. J. Biomed. Sci..

[25] Rotunno M, Hu N, Su H (2011). A gene expression signature from peripheral whole blood for stage I lung adenocarcinoma. Cancer Prev. Res. (Phila.).

[26] Baylin, S.B., Jones, P.A. Epigenetic determinants of cancer. *Cold Spring Harbor Perspect. Biol*. **8**(9), a019505. 10.1101/cshperspect.a019505 (2016). 10.1101/cshperspect.a019505 PMC5008069 27194046

[27] Irizarry RA, Ladd-Acosta C, Wen B (2009). The human colon cancer methylome shows similar hypo- and hypermethylation at conserved tissue-specific CpG island shores. Nat. Genet..

[28] Baylin SB, Jones PA (2011). A decade of exploring the cancer epigenome—Biological and translational implications. Nat. Rev. Cancer.

[29] Zhang J, Luo L, Dong J (2020). A prognostic 11-DNA methylation signature for lung squamous cell carcinoma. J. Thorac. Dis..

[30] Peng Y, Wu Q, Wang L (2020). A DNA methylation signature to improve survival prediction of gastric cancer. Clin. Epigenet..

[31] Liew PL, Fang CY, Lee YC (2016). DEF6 expression in ovarian carcinoma correlates with poor patient survival. Diagn. Pathol..

[32] Liu J, Li H, Shen S (2018). Alternative splicing events implicated in carcinogenesis and prognosis of colorectal cancer. J. Cancer.

[33] Zhang Y, Zhu H, Zhang X (2013). Clinical significance of MYT1L gene polymorphisms in Chinese patients with gastric cancer. PLoS ONE.

[34] Liu SM, Lin CH, Lu J (2018). miR-596 modulates melanoma growth by regulating cell survival and death. J. Invest. Dermatol..

[35] Xiang T, Yu F, Fei R (2016). CHRNA7 inhibits cell invasion and metastasis of LoVo human colorectal cancer cells through PI3K/Akt signaling. Oncol. Rep..

[36] Dai W, Zhou J, Wang H (2020). miR-424-5p promotes the proliferation and metastasis of colorectal cancer by directly targeting SCN4B. Pathol. Res. Pract..

[37] Yuan L, Hu F, Zhang Y (2020). Identification and functional analysis of a novel splice variant of AC3-33 in breast cancer. Exp. Ther. Med..

[38] Kai Y, Amatya VJ, Kushitani K (2019). Mucin 21 is a novel, negative immunohistochemical marker for epithelioid mesothelioma for its differentiation from lung adenocarcinoma. Histopathology.

[39] Colaprico A, Silva TC, Olsen C (2016). TCGAbiolinks: An R/Bioconductor package for integrative analysis of TCGA data. Nucleic Acids Res..

[40] Friedman J, Hastie T, Tibshirani R (2010). Regularization paths for generalized linear models via coordinate descent. J. Stat. Softw..

[41] Pidsley R, Wong CCY, Volta M (2013). A data-driven approach to preprocessing Illumina 450K methylation array data. BMC Genom..

[42] Aryee MJ, Jaffe AE, Corrada-Bravo H (2014). Minfi: a flexible and comprehensive Bioconductor package for the analysis of Infinium DNA methylation microarrays. Bioinformatics (Oxford, England)..

[43] De Angelis G, De Angelis R, Frova L (1994). MIAMOD: A computer package to estimate chronic disease morbidity using mortality and survival data. Comput. Methods Programs Biomed..

[44] Robin X, Turck N, Hainard A (2011). pROC: An open-source package for R and S+ to analyze and compare ROC curves. BMC Bioinform..

[45] Harrell FE (2015). Regression Modeling Strategies: With Applications to Linear Models, Logistic Regression, and Survival Analysis.

