# Supplementary figures and images for: Identification of a methylomics-associated nomogram for predicting overall survival of stage I–II lung adenocarcinoma
Source: Sci Rep. 2021 May 11;11:9938. doi: 10.1038/s41598-021-89429-4 (PMC8113535; doi:10.1038/s41598-021-89429-4)

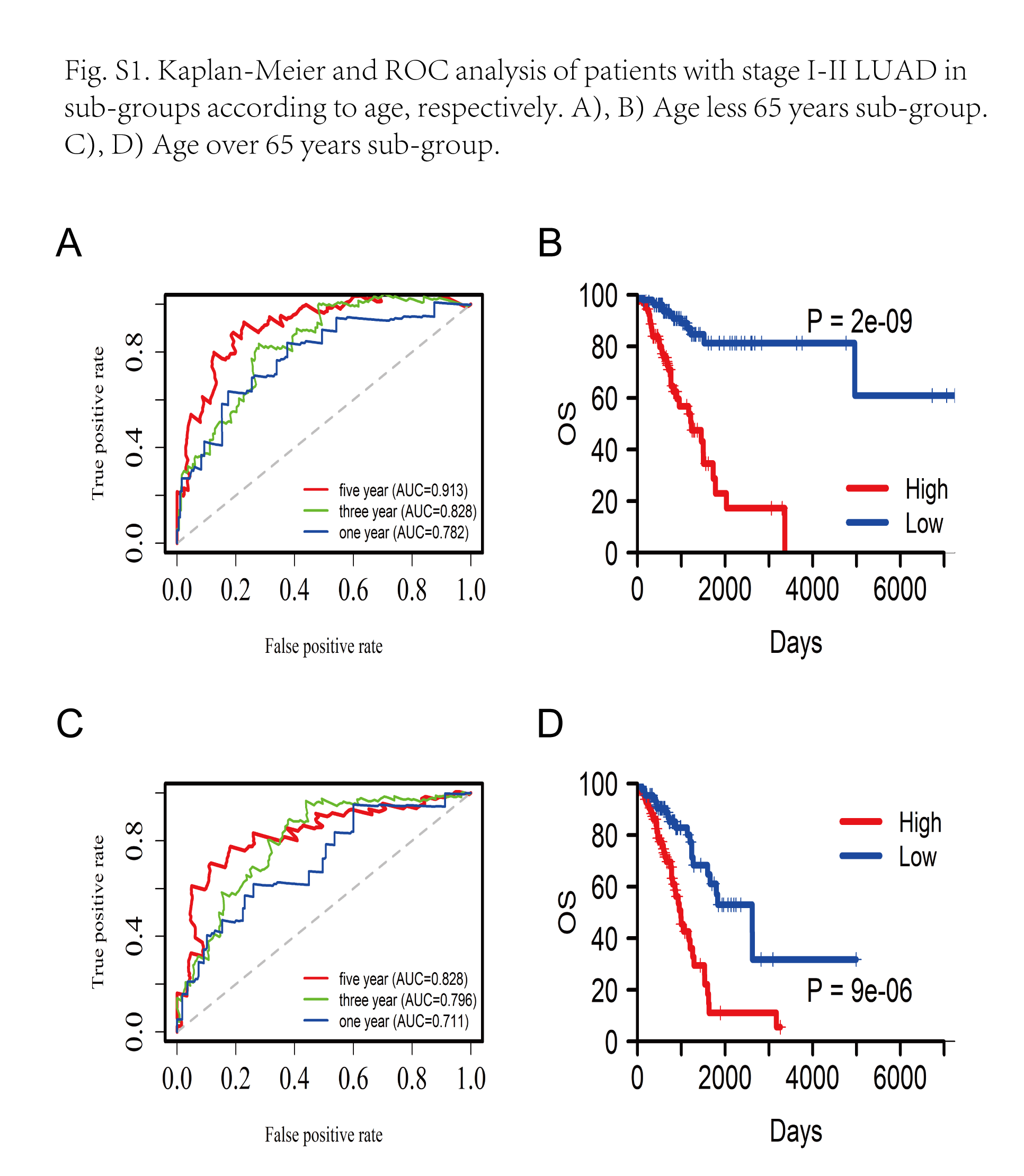

Supplement: Supplementary file 1 — Supplementary Figure S1. [file 41598_2021_89429_MOESM1_ESM.tif]

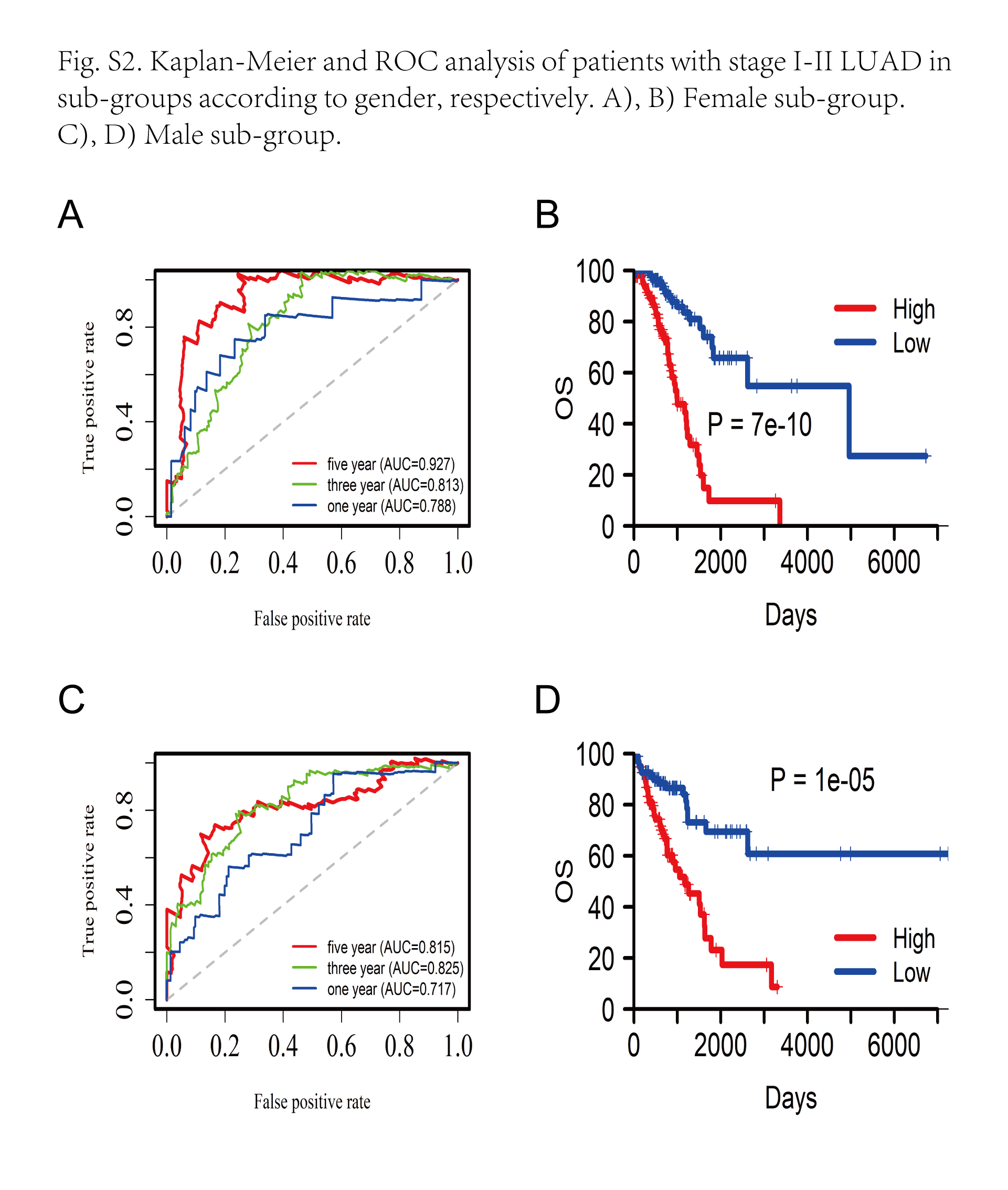

Supplement: Supplementary file 2 — Supplementary Figure S2. [file 41598_2021_89429_MOESM2_ESM.tif]

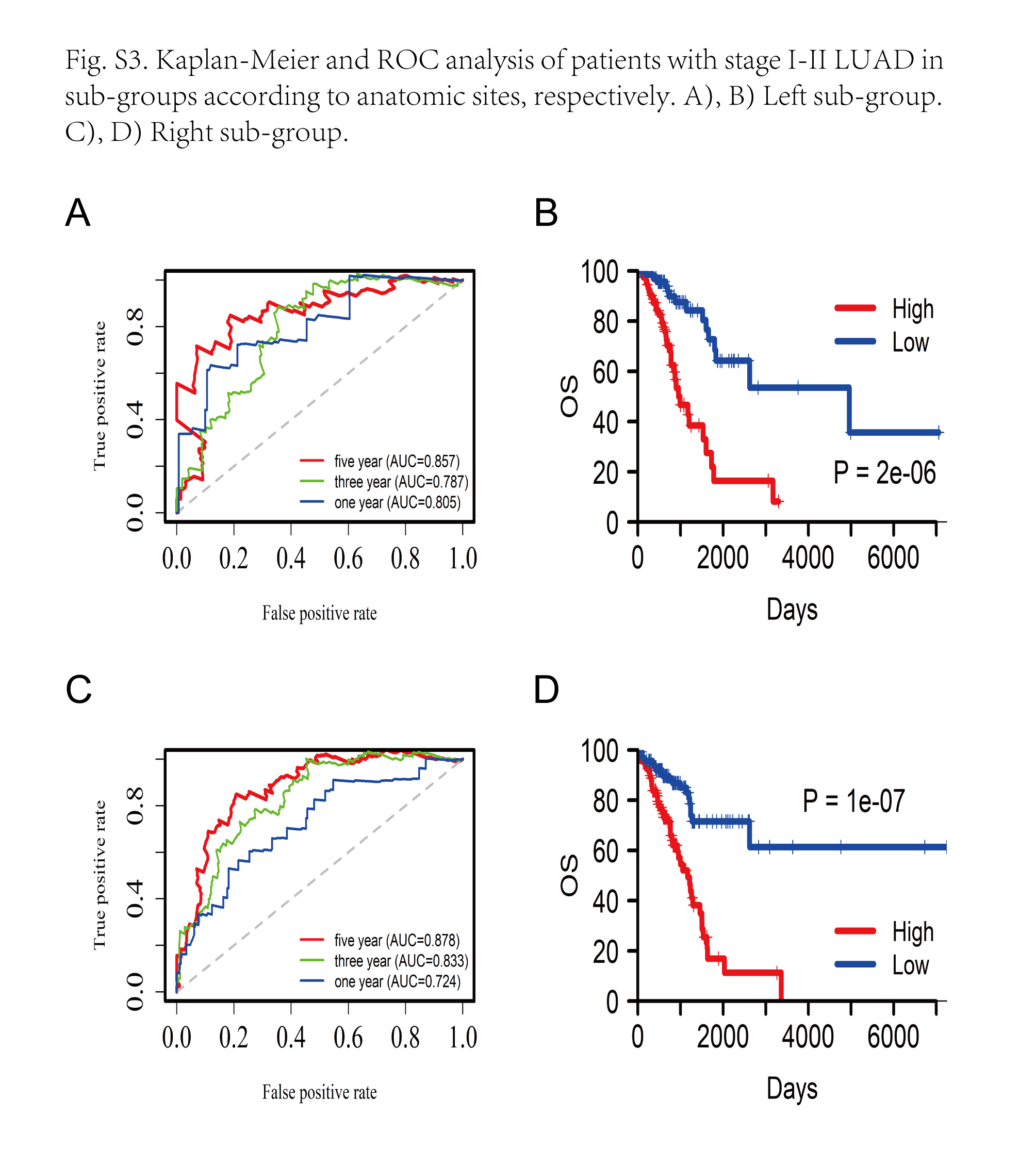

Supplement: Supplementary file 3 — Supplementary Figure S3. [file 41598_2021_89429_MOESM3_ESM.tif]

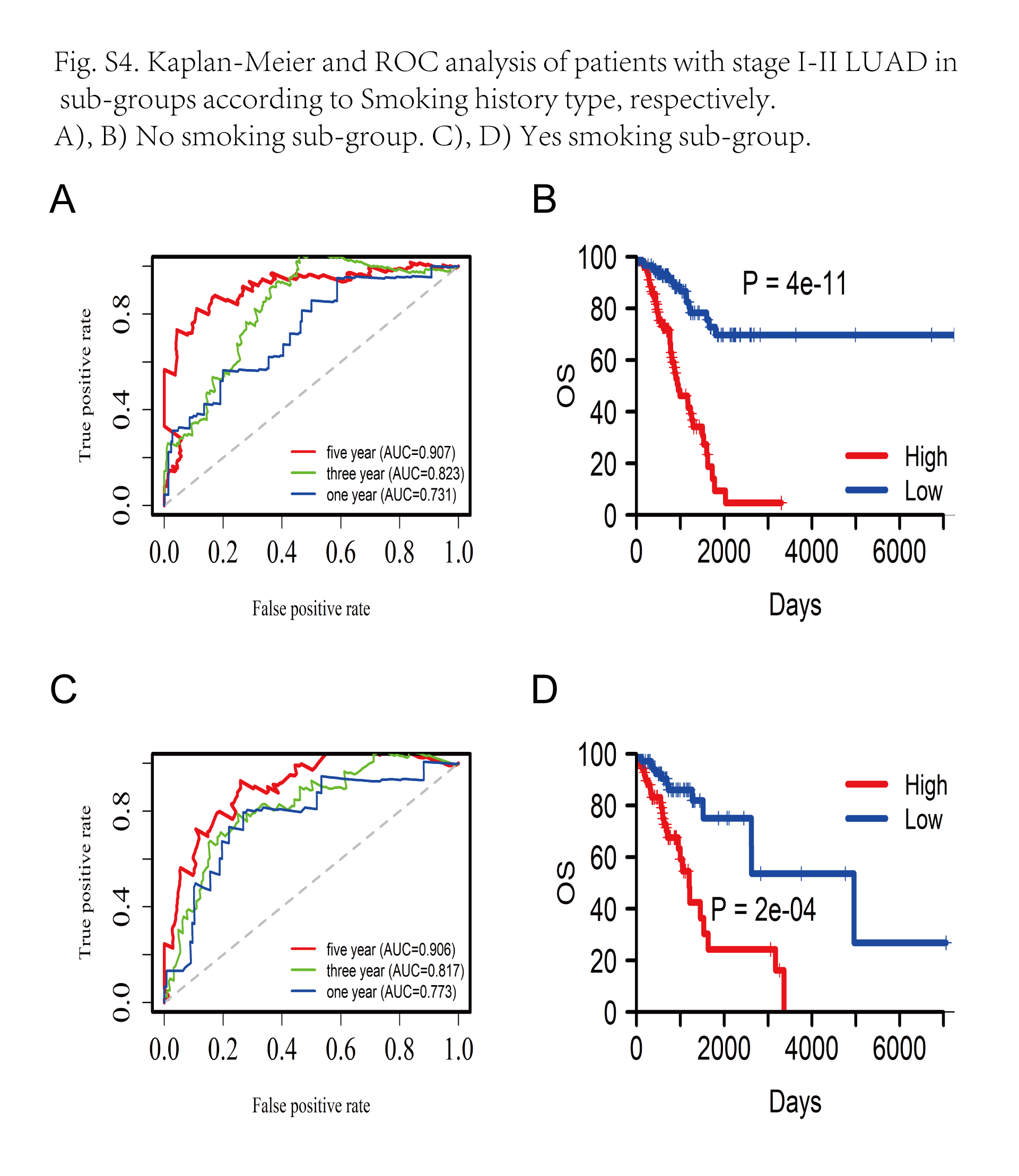

Supplement: Supplementary file 4 — Supplementary Figure S4. [file 41598_2021_89429_MOESM4_ESM.tif]

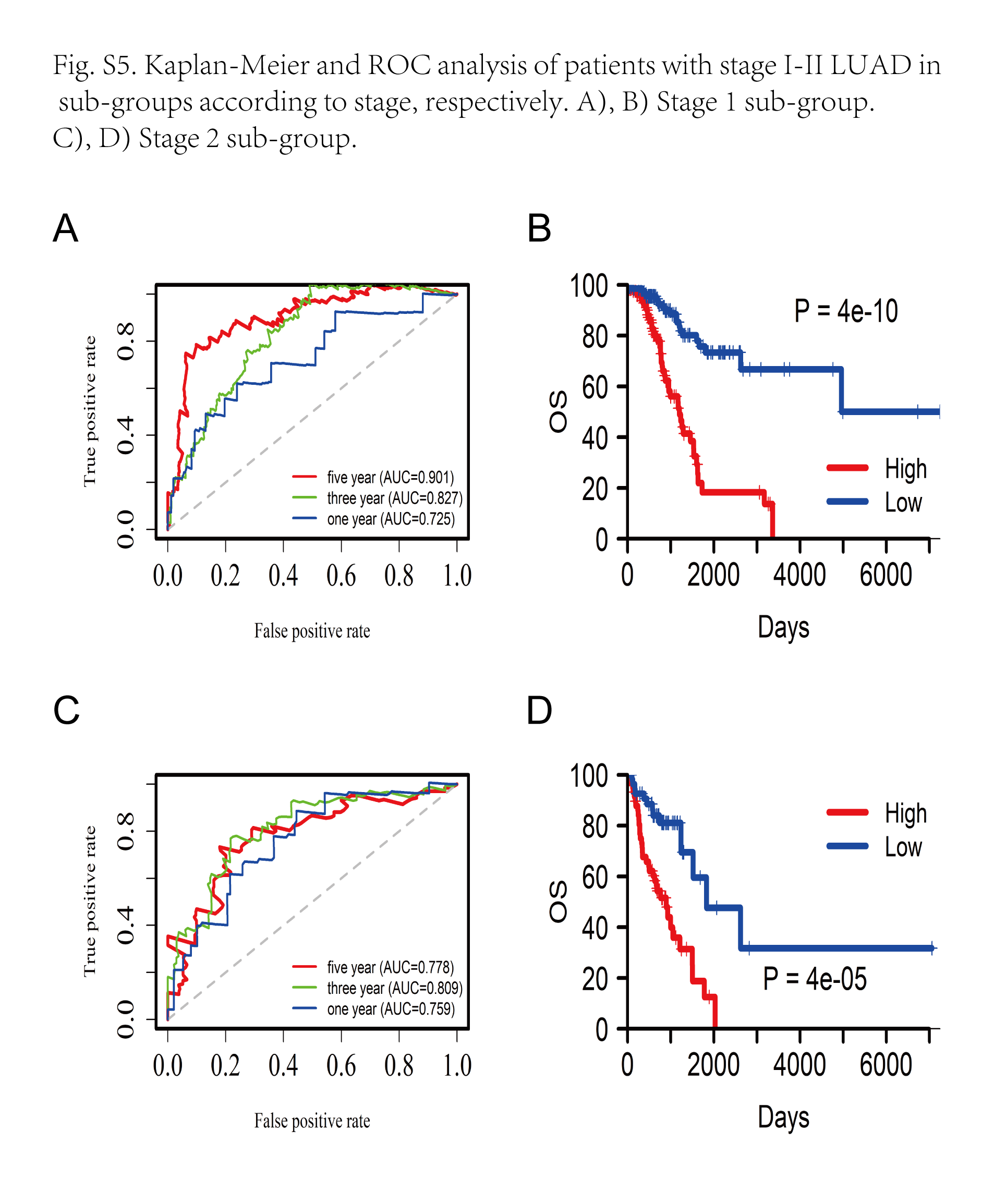

Supplement: Supplementary file 5 — Supplementary Figure S5. [file 41598_2021_89429_MOESM5_ESM.tif]
